# The relationship between mean corpuscular hemoglobin concentration and mortality in hypertensive individuals: A population-based cohort study

**DOI:** 10.1371/journal.pone.0301903

**Published:** 2024-05-09

**Authors:** Dan Li, Qing Zhang, Zhishen Ruan, Yue Zhang, Xiaohe Liu, Guihong Zhang, Hengyi Zhao, Jing Li, Bo Wu

**Affiliations:** 1 The First Clinical College, Shandong Chinese Medical University, Jinan, People’s Republic of China; 2 Department of Emergency, Dongying People’s Hospital, Donging, People’s Republic of China; 3 Department of Cardiovascular Medicine, The First Affiliated Hospital of Shandong University of Traditional Chinese Medicine, Jinan, People’s Republic of China; 4 The First Affiliated Hospital of Shandong First Medical University, Jinan, People’s Republic of China; Xi’an Jiaotong University, CHINA

## Abstract

**Introduction:**

Hematology is an essential field for investigating the prognostic outcomes of cardiovascular diseases (CVDs). Recent research has suggested that mean corpuscular hemoglobin concentration (MCHC) is associated with a poor prognosis in several CVDs. There is no evidence of a correlation between MCHC and hypertension. Therefore, our study aimed to analyze the association of MCHC with all-cause and cardiovascular mortality in hypertensive patients.

**Methods:**

We used cohort data from U.S. adults who participated in the National Health and Nutrition Examination Survey from 1999–2014. COX regression was applied to analyze the relationship between MCHC and all-cause and cardiovascular mortality. In addition, three models were adjusted to reduce confounding factors. We reanalyzed the data after propensity score matching (PSM) to inspect the stability of the results. Stratified analysis was additionally adopted to investigate the results of each subgroup.

**Results:**

Our research included 15,154 individuals. During a mean follow-up period of 129 months, 30.6% of the hypertensive population succumbed to mortality. Based on previous studies, we categorized patients with MCHC ≤33mg/dl as the hypochromia group and those with >33mg/dl as the non-hypochromia group. After PSM, the hypochromia group had higher all-cause mortality (adjusted hazard ratio [HR]:1.26, 95% confidence interval [95%CI]:1.11–1.43) and cardiovascular mortality (adjusted HR:1.42, 95%CI:1.12–1.80) than the non-hypochromia group. The results of the COX regression remain stable after matching. Stratified analyses before PSM revealed an interaction of anemia in the relationship between MCHC and mortality, whereas there was no significant interaction after matching.

**Conclusion:**

In hypertensive individuals, low MCHC was correlated with a poor prognosis. Further studies on MCHC are necessary to analyze the potential mechanisms of its poor prognosis in hypertensive populations.

## Introduction

Hypertension, a condition progressively damages target organs, stands as a primary cause of disability and premature death worldwide [1]. The prevalence of hypertension continues to escalate, reaching 31.5% in 2010 [2,3]. Moreover, the economic burden of hypertension has become an enormous challenge to global health care. It is well known that obesity with a body mass index >30 kg/m^2^ and smoking are associated with mortality in hypertensive patients [4–6]. The identification of additional risk factors can contribute to a comprehensive exploration of the potential pathogenesis and early intervention in mortality outcomes related to hypertension. The pathological changes in hypertension and its comorbidities are mainly stress responses to metabolic and hemodynamic factors [7]. Therefore, hematology is an important area of investigation in the prognostic prediction studies of hypertension.

The mean hemoglobin level in red blood cells is defined as mean corpuscular hemoglobin concentration (MCHC), which is a proxy for the amount of oxygen-carrying capacity and iron content [8,9]. Low MCHC levels imply the presence of hypochromia, a clinical event capable of affecting ventricular diastolic dysfunction and large artery remodeling [10,11]. Additionally, low MCHC may be associated with inflammatory immunity, iron imbalance, and erythrocyte release disorders. Recent data have suggested that MCHC correlates with cardiopulmonary disease prognosis with acute pulmonary embolism [12], acute myocardial infarction [13], and heart failure [14]. The relationship between MCHC and the prognosis of hypertension deserves further exploration. Therefore, we plan to use the National Health and Nutrition Examination Survey (NHANES) to investigate the association of MCHC with all-cause and cardiovascular mortality in hypertensive populations.

## Materials and methods

### Study population

The NHANES database is derived from a sample representing the entire U.S. population. The data collection includes a standardized home interview, physical examination, and biological specimen. NHANES database was approved by the National Center for Health Statistics Research Ethics Review Board, and all patients had signed informed consent [15]. It is important to note that all participants in the database remain de-identified to ensure privacy. Our study adheres to the criteria for Strengthening Observational Studies in Epidemiology [16]. All statistics used in the study were extracted from https://www.cdc.gov/nchs/nhanes.

We collected and analyzed participants recorded through NHANES from 1999–2014. After excluding missing data and people ≤20 years old, 15,154 hypertensive patients were included in our research. People with one or more of the following three conditions are defined as hypertensive: previously diagnosed as hypertensive by a clinician, taking blood pressure-lowering medications, and systolic blood pressure (SBP) ≥ 140 mmHg or diastolic blood pressure (DBP) ≥ 90 mmHg [17]. According to the critical value of MCHC, we defined patients ≤33mg/dl as the hypochromia group and those >33mg/dl as the non-hypochromia group [18].

**Fig 1:**
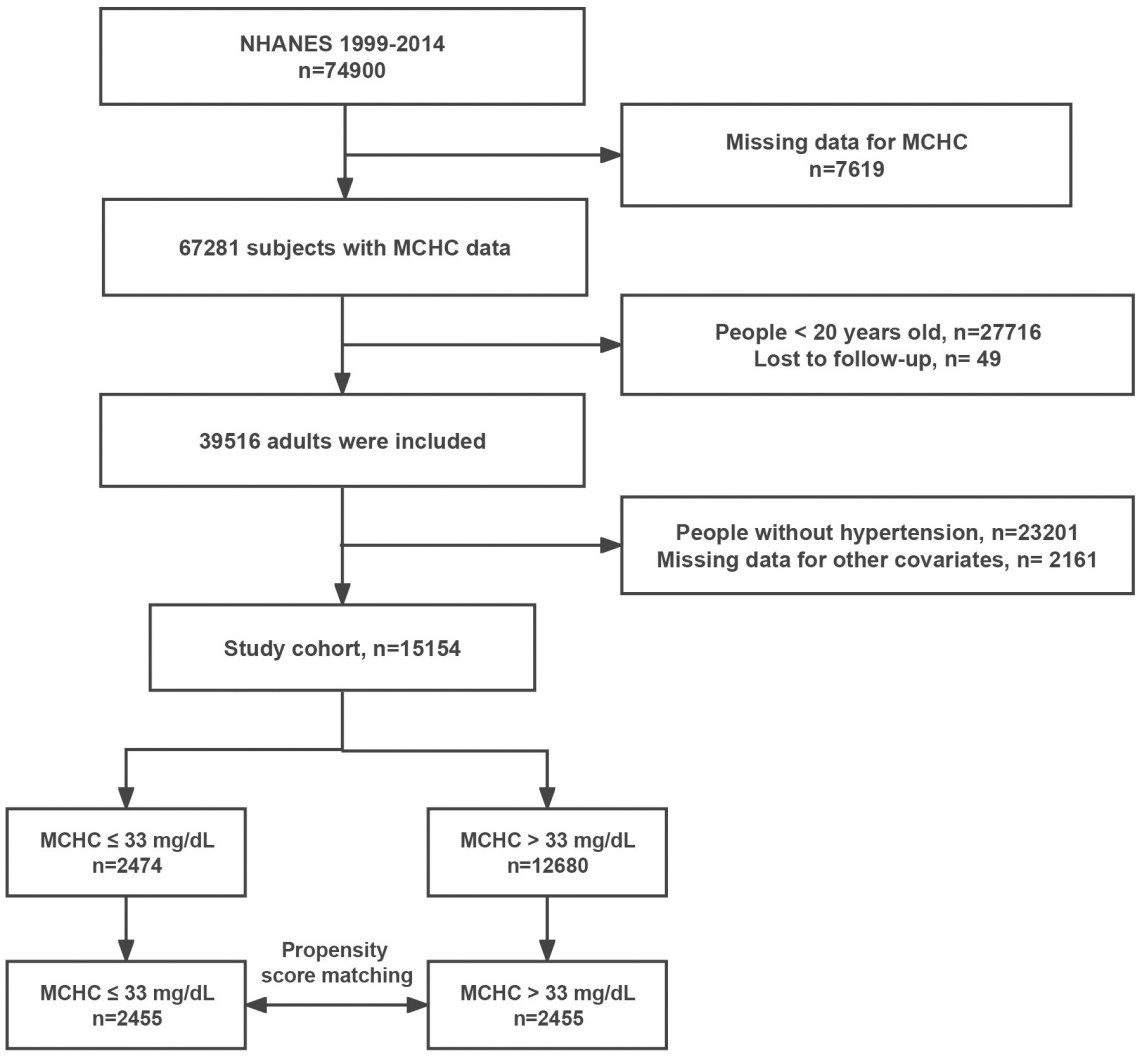
Flow chart.

Fig 1 illustrates the detailed inclusion and exclusion criteria.

### Study outcomes

All-cause and cardiovascular mortality in hypertensive individuals during the follow-up period were the primary outcomes of the study. In the public access file, the National Death Index linked to NHANES contains mortality distribution status and cause of death statistics. See additional documents for details on the definition of cardiovascular mortality.

### Study covariates

We incorporated demographic data (race, gender, age, education, smoking status, body mass index [BMI], and marital status) derived from self-reported data. The race was divided into Non-Hispanic White, Mexican American, Non-Hispanic Black, and other races. BMI was classified into three levels (<25, 25–30, and >30 kg/m^2^). We categorized smoking status as never (smoking less than 100 cigarettes in a lifetime), previous (smokes more than 100 cigarettes but has quit), and current. Marital status is distinguished as married and single.

In addition, the patient’s self-reported medical history (chronic kidney disease [CKD], cardiovascular disease [CVD], diabetes, hyperlipidemia, chronic obstructive pulmonary disease [COPD], and anemia) was considered comorbidities. Definitions of the above comorbidities are detailed in the additional document.

### Statistical analysis

In descriptive statistics, t-tests or non-parametric Mann-Whitney U tests were used to compare baseline characteristics of continuous variables. Kruskal Wallis and Chi-square (or Fisher’s exact) tests to compare categorical variables. COX regression was applied to analyze the association of both groups with all-cause and cardiovascular mortality. Furthermore, we adjusted for potential bias using three models. Model 1 adjusted race, sex, and age. Model 2 adjusted model 1 plus BMI, smoking, education, and marriage. Model 3 adjusted model 2 plus diabetes, hyperlipidemia, CKD, CVD, COPD, and anemia. We performed a stratified analysis by race, sex, age, smoking status, BMI, diabetes, cardiovascular disease, and anemia, presenting the results in a forest plot. Due to the large difference in numbers between the two MCHC subgroups, with a ratio of approximately 1:5 people, we implemented propensity score matching (PSM) to minimize bias [19,20]. ^P^SM used a 1:1 nearest neighbor matching algorithm and set the caliper value to 0.01. The selected confounders for matching included age, gender, race, education, marital status, BMI, smoking, and comorbidities (CVD, CKD, diabetes, hyperlipidemia, COPD, anemia).

All data were statistically analyzed using R v4.1.3 (http://www.R-project.org, The R Foundation).

## Results

### Baseline characteristics of participants

According to the critical value of MCHC, we separated participants into hypochromia and non-hypochromia groups [18]. Table 1 illustrates the demographic characteristics of participants before propensity score matching (PSM). Among 15,154 participants, 2,474 were assigned to the hypochromia group, and 12,680 to the non-hypochromia group. The hypochromia group had an average age of 58.0 years old, of whom 58.0% were female. The hypochromia group had higher age and BMI, were more likely to have never smoked, and had comorbidities of CVD, CKD, diabetes, hyperlipidemia, and anemia. Over the follow-up period, mortality was significantly higher in the hypochromia group compared to the non-hypochromia group (31.0% vs. 23.6%).

**Table 1:** Baseline characteristics of patients with hypertension before PSM.

Variables	MCHC ≤ 33 mg/dL N = 2474	MCHC > 33 mg/dL N = 12680	*P*
**Age, year**	58.5 ± 0.45	56.6 ± 0.25	< 0.001
**Female, n (%)**	1349 (58.0)	6320 (50.4)	< 0.001
**Race, n (%)**			< 0.001
White	691 (48.7)	6743 (76.0)	
Mexican	243 (4.5)	1974 (5.5)	
Black	1267 (36.5)	2399 (9.6)	
Other Races	273 (10.4)	1564 (9.0)	
**Education, n (%)**			< 0.001
< college	1466 (53.2)	7118 (47.0)	
≥ College	1008 (46.8)	5562 (53.0)	
**BMI, n (%)**			0.004
< 25	516 (21.2)	2639 (19.9)	
25–30	732 (29.4)	4440 (34.7)	
> 30	1226 (49.5)	5601 (45.4)	
**Smoke, n (%)**			0.015
Never smoker	1300 (52.3)	6195 (48.6)	
Former smoker	769 (31.2)	4085 (32.0)	
Current smoker	405 (16.5)	2400 (19.4)	
**Marital status, n (%)**			< 0.001
Married	1339 (58.9)	7640 (65.0)	
Single	1135 (41.1)	5040 (35.0)	
**Comorbidities, n (%)**			
CVD	577 (21.0)	2533 (16.9)	< 0.001
CKD	927 (33.6)	3939 (25.1)	< 0.001
Diabetes	846 (28.0)	3443 (21.9)	< 0.001
Hyperlipidemia	496 (19.5)	2168 (16.5)	0.009
COPD	171 (7.1)	820 (6.5)	0.492
Anemia	631 (23.1)	1105 (6.0)	< 0.001
**Status, n (%)**			< 0.001
Alive	1616 (69.0)	8895 (76.4)	
Death	858 (31.0)	3785 (23.6)	

PSM: Propensity score matching; BMI: Body mass index; CVD: Cardiovascular disease; CKD: Chronic kidney disease. COPD: Chronic obstructive pulmonary disease.

### Correlation analysis of MCHC and mortality before and after matching

The follow-up period was 129 months before matching and 120 months after matching. Before matching, the hazard ratio (HR) for all-cause mortality in the hypochromia group was 1.21 (95% confidence interval [95% CI]: 1.09,1.35) after adjustment for model 3. When MCHC was treated as a continuous variable, the HR was 0.93 (95% CI: 0.89,0.98). For cardiovascular mortality, HR was 1.44 (95% CI: 1.22,1.71) in the hypochromia group and 0.84 (95% CI: 0.78,0.91) in the continuous MCHC (Table 2).

**Table 2:** Weighted association between MCHC and mortality before PSM.

	Unadjusted	Model 1	Model 2	Model 3
**All-cause Mortality**				
Deaths, No. (%)	4643 (30.6%)			
MCHC, continuous	0.85 (0.81,0.89) ^‡^	0.90 (0.86,0.95) ^‡^	0.90 (0.86,0.95) ^‡^	0.93 (0.89,0.98) ^‡^
> 33 mg/dL	1(Ref)	1(Ref)	1(Ref)	1(Ref)
≤ 33 mg/dL	1.42 (1.27,1.59) ^‡^	1.33 (1.20,1.48) ^‡^	1.34 (1.21,1.48) ^‡^	1.21 (1.09,1.35) ^‡^
**CVD Mortality**				
Deaths, No. (%)	1303 (8.6%)			
MCHC, continuous	0.76 (0.71,0.82) ^‡^	0.81 (0.74,0.87) ^‡^	0.81 (0.75,0.87) ^‡^	0.84 (0.78,0.91) ^‡^
> 33 mg/dL	1(Ref)	1(Ref)	1(Ref)	1(Ref)
≤ 33 mg/dL	1.74 (1.47,2.05) ^‡^	1.62 (1.37,1.90) ^‡^	1.61 (1.37,1.90) ^‡^	1.44 (1.22,1.71) ^‡^

Data are presented as HR (95%CI).

Model 1: Adjust for age, sex, and eth.

Model 2: Adjust for age, sex, eth, BMI, smoke, education, and married.

Model 3: Adjust for age, sex, eth, BMI, smoke, education, diabetes, hyperlipidemia, CKD, CVD, COPD, Anemia.

^†^P < 0.05.

^‡^P < 0.01.

Table 3 displays the demographic characteristics after PSM, with P values for baseline differences between the two groups after matching exceeding 0.05. After matching, the hypochromic group showed a 26% increased risk of all-cause mortality and a 42% increased risk of cardiovascular death (Table 4). The correlation between MCHC and mortality remained stable in the three models before and after matching.

**Table 3:** Baseline characteristics of patients with hypertension after PSM.

Variables	MCHC ≤ 33 mg/dL N = 2455	MCHC > 33 mg/dL N = 2455	*P*
**Age, year**	58.1± 0.45	58.5± 0.43	0.129
**Female, n (%)**	1335 (57.9)	1310 (55.9)	0.286
**Race, n (%)**			0.742
White	691 (49.0)	640 (47.6)	
Mexican	243 (4.5)	254 (5.3)	
Black	1248 (36.0)	1251 (36.1)	
Other Race	273 (10.4)	310 (11.1)	
**Education, n (%)**			0.814
< college	1457 (53.2)	1453 (52.8)	
≥ College	998 (46.8)	1002 (47.2)	
**BMI, n (%)**			0.998
< 25	515 (21.3)	527 (21.4)	
25–30	727 (29.4)	769 (29.2)	
> 30	1213 (49.3)	1159 (49.4)	
**Smoke, n (%)**			0.892
Never smoker	1290 (52.2)	1289 (53.1)	
Former smoker	761 (31.2)	752 (30.5)	
Current smoker	404(16.6)	414(16.4)	
**Marital status, n (%)**			0.974
Married	1126 (41.0)	1123 (41.1)	
Single	1329 (59.0)	1332 (58.9)	
**Comorbidities, n (%)**			
CVD	571 (21.0)	554 (19.6)	0.343
CKD	918 (33.6)	914 (32.8)	0.664
Diabetes	839 (28.0)	810 (28.4)	0.791
Hyperlipidemia	490 (19.4)	501 (18.8)	0.681
COPD	168 (7.0)	186 (7.7)	0.473
Anemia	612 (22.6)	596 (19.9)	0.076
**Status, n (%)**			0.002
Alive	1601 (69.0)	1706 (74.4)	
Death	854 (31.0)	749 (25.6)	

PSM: Propensity score matching; BMI: Body mass index; CVD: Cardiovascular disease; CKD: Chronic kidney disease. COPD: Chronic obstructive pulmonary disease.

**Table 4:** Weighted association between MCHC and mortality after PSM.

	Unadjusted	Model 1	Model 2	Model 3
**All-cause Mortality**				
Deaths, No. (%)	1603 (32.6)			
MCHC, continuous	0.90 (0.85,0.97) ^‡^	0.90 (0.84,0.96) ^‡^	0.90 (0.84,0.96) ^‡^	0.91 (0.85,0.97) ^‡^
> 33 mg/dL	1(Ref)	1(Ref)	1(Ref)	1(Ref)
≤ 33 mg/dL	1.27 (1.11,1.46) ^‡^	1.23 (1.09,1.40) ^‡^	1.23 (1.10,1.39) ^‡^	1.26 (1.11,1.43) ^‡^
**CVD Mortality**				
Deaths, No. (%)	478 (9.7)			
MCHC, continuous	0.88 (0.79,0.98) ^†^	0.87 (0.77,0.98) ^†^	0.87 (0.77,0.98) ^†^	0.88 (0.78,0.98) ^†^
> 33 mg/dL	1(Ref)	1(Ref)	1(Ref)	1(Ref)
≤ 33 mg/dL	1.42 (1.13,1.79) ^‡^	1.38 (1.10,1.74) ^‡^	1.38 (1.10,1.74) ^‡^	1.42 (1.12,1.80) ^‡^

Data are presented as HR (95%CI).

Model 1: Adjust for age, sex, and eth.

Model 2: Adjust for age, sex, eth, BMI, smoke, education, and married.

Model 3: Adjust for age, sex, eth, BMI, smoke, education, diabetes, hyperlipidemia, CKD, CVD, COPD, Anemia.

^†^P < 0.05.

^‡^P < 0.01.

### Stratification analysis before and after PSM

**Fig 2:**
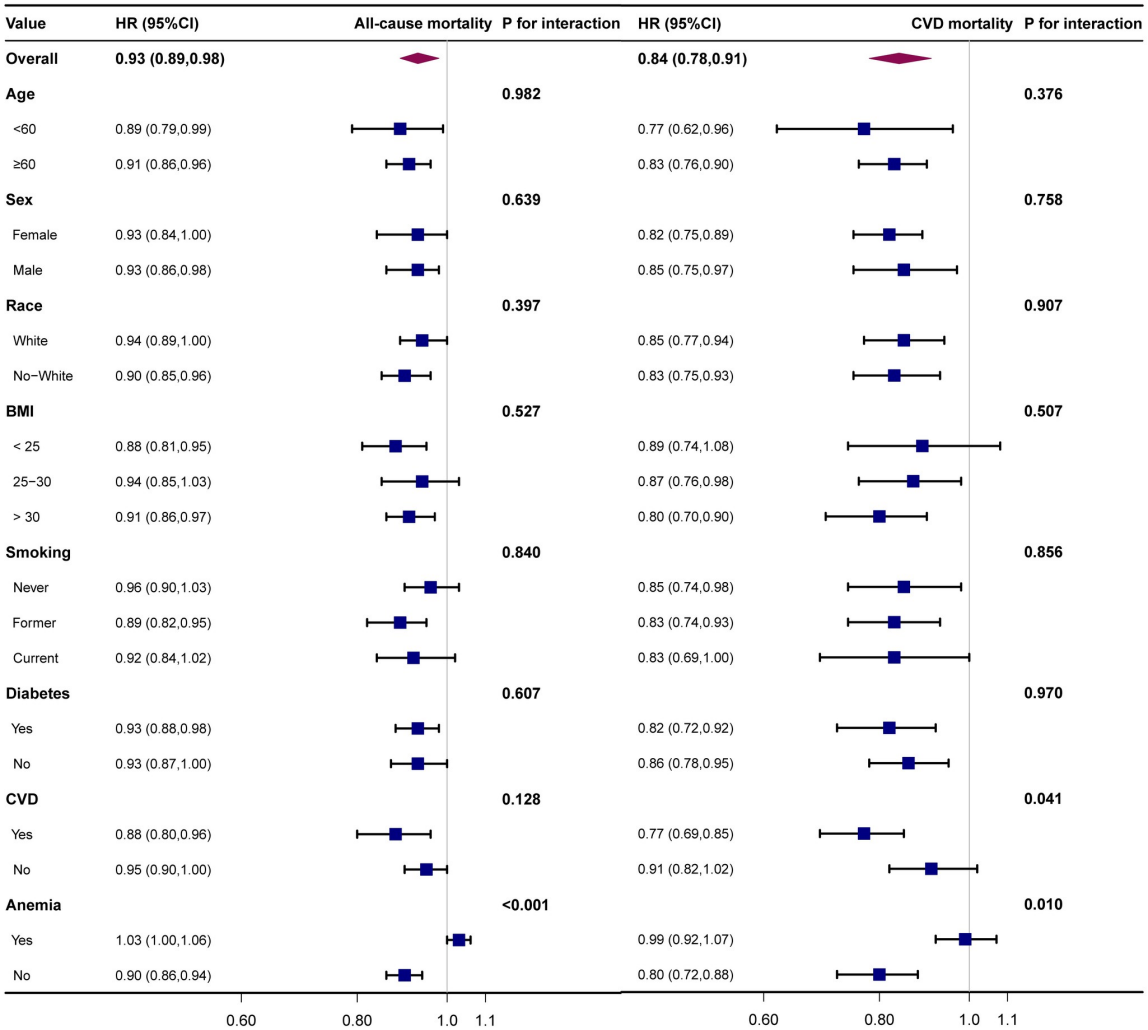
Stratified analysis of MCHC and mortality before PSM.

**Fig 3:**
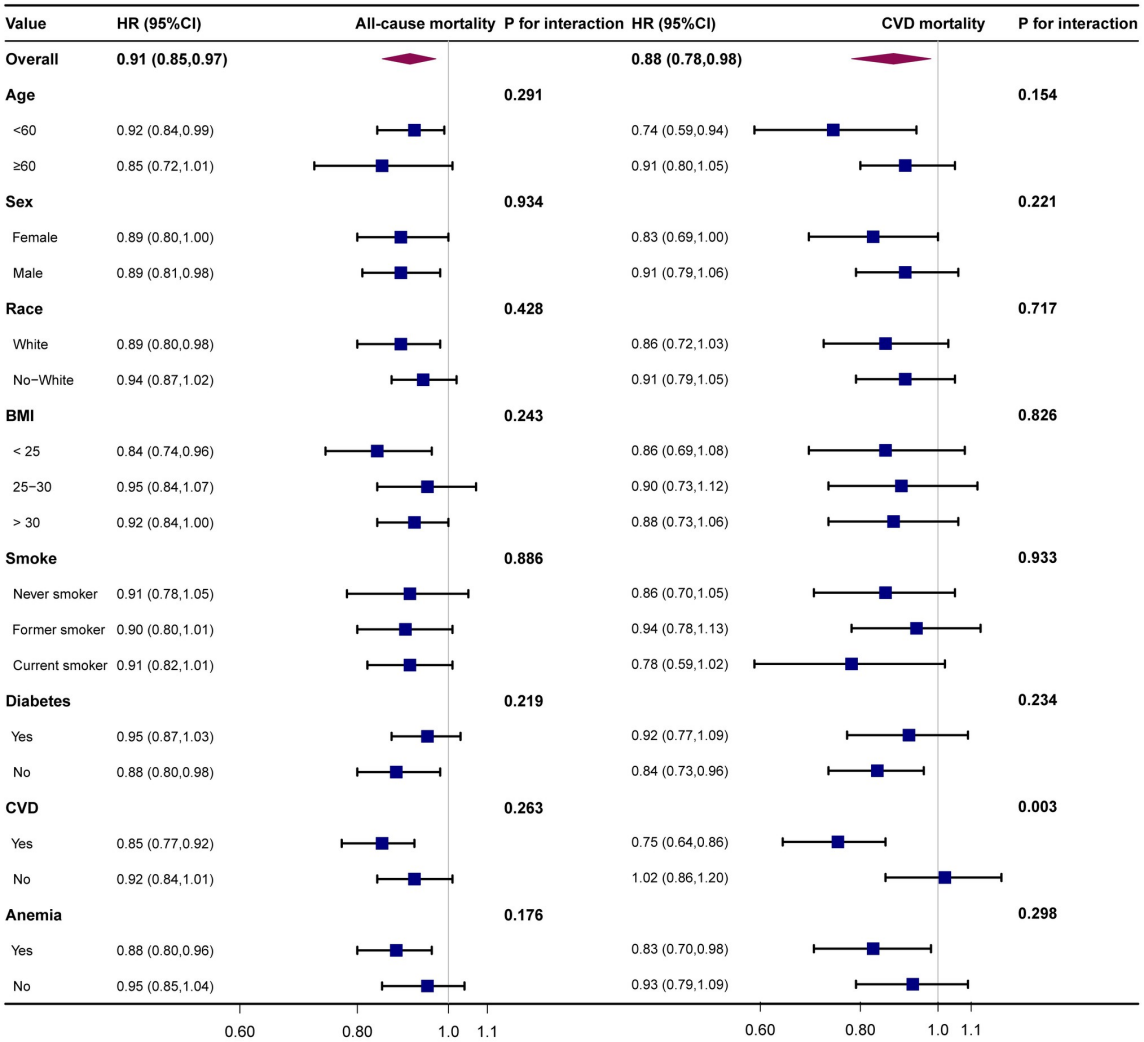
Stratified analysis of MCHC and mortality after PSM.

We applied a stratified analysis to explore the results’ stability in different populations. There was no apparent difference in the correlation between MCHC and mortality when age, sex, race, BMI, smoking, and diabetes were stratified. Stratified analyses before PSM revealed an interaction of anemia in the relationship between MCHC and mortality (Fig 2), whereas there was no significant interaction after matching (Fig 3). In cardiovascular mortality, P for interaction <0.05 for combined CVD, regardless of before and after pairing.

## Discussion

To our knowledge, this research is the first to find that a low level of MCHC is independently related to poor prognosis in hypertension. Significantly, all-cause and cardiovascular mortality were higher in the hypochromia group than in the non-hypochromia group before and after PSM.

Studies on the correlation between MCHC and the prognosis of various diseases have been increasing. Previous studies have revealed that low levels of MCHC serve as an independent risk factor for acute myocardial infarction and stroke [13,21]. Ruan identified a connection between low MCHC levels and poor prognosis in patients with acute pulmonary embolism [12]. In cancer, overall survival after hepatectomy for hepatocellular carcinoma was shorter in patients with low MCHC (HR = 0.372) [22]. Similarly, Qu noted that MCHC was an independent predictive factor for non-small cell lung cancer recurrence-free survival and overall survival [23].

MCHC indicates hemoglobin concentration in red blood cells, often regarded as a marker of anemia. In our study, pre-match stratified analysis displayed an interactive effect of anemia on mortality in hypertensive patients. Patients with poorly controlled blood pressure tend to have comorbid anemia [24], and these patients have worse cardiovascular mortality outcomes on the survival curve [25]. Studies have suggested that anemia is independently associated with high pulse pressure, possibly due to increased SBP and decreased DBP due to atherosclerosis [10]. Anemia leads to increased cardiac load, impaired ventricular systolic function, and activation of the sympathetic nervous system, which leads to cardiac remodeling and impaired myocardial function [26,27].

Hosseinpour’s findings indicated that among the erythrocyte variables studied (red blood cell distribution width, hemoglobin, hematocrit, MCHC), MCHC emerged as the most sensitive predictor for prognosis in heart failure [14]. Erythrocytes play a crucial role as the primary cellular component of blood, participating in hypoxic vasodilatory mechanisms through NO metabolism and ATP release [28,29]. Most hypertensive patients have higher blood viscosity than healthy people; high viscosity, low red blood cell deformability, and increased microvascular resistance promote increased peripheral resistance and arterial blood pressure [30], leading to or worsening hypertension [31]. Fornal found that low levels of MCHC were accompanied by elevated IL-6 [32]. In the inflammatory response, cell-induced immune effector mechanisms produce the cytokine IL-6 [33]. IL-6, in turn, has dual effects by inducing ferritin expression while inhibiting iron absorption in the duodenum. A decrease in MCHC34 manifests the disruption of iron homeostasis in hypertensive patients [34].

The strengths of this study are the long follow-up period and the large sample size. In addition, we can reduce the effect of confounders through PSM and model adjustment. Moreover, incorporating complex sampling weighting enables the results to reflect the entire U.S. population. However, there are limitations to our research. First, this study is monocentric and inevitably has the limitations of a geographical analysis. Second, MCHC values are collected at the beginning of follow-up, and a dynamic MCHC in the future is more helpful in assessing the predictive risk of hypertensive patients.

## Conclusion

In hypertensive individuals, low MCHC was correlated with a poor prognosis. Further research on MCHC is necessary to identify high-risk populations and to investigate its potential mechanisms in hypertension.

## Supporting information

S1 Data Original data. (XLSX)
